# Identification of intelligence-related proteins through a robust two-layer predictor

**DOI:** 10.1080/19420889.2022.2143101

**Published:** 2022-11-15

**Authors:** Aida Shomali, Mohammad Sadegh Vafaei Sadi, Mohammad Reza Bakhtiarizadeh, Sasan Aliniaeifard, Anthony Trewavas, Paco Calvo

**Affiliations:** a Department of Horticulture, College of Aburaihan, University of Tehran, Tehran, Iran; b Department of Animal and Poultry Science, College of Aburaihan, University of Tehran, Tehran, Iran; c School of Biological Sciences, Institute of Molecular Plant Science, University of Edinburgh, UK; d Minimal Intelligence Lab, University of Murcia, Spain

**Keywords:** Intell_pred, protein features, support vector machine, learning

## Abstract

In this study, we advance a robust methodology for identifying specific intelligence-related proteins across phyla. Our approach exploits a support vector machine-based classifier capable of predicting intelligence-related proteins based on a pool of meaningful protein features. For the sake of illustration of our proposed general method, we develop a novel computational two-layer predictor, Intell_Pred, to predict query sequences (proteins or transcripts) as intelligence-related or non-intelligence-related proteins or transcripts, subsequently classifying the former sequences into learning and memory-related classes. Based on a five-fold cross-validation and independent blind test, Intell_Pred obtained an average accuracy of 87.48 and 88.89, respectively. Our findings revealed that a score >0.75 (during prediction by Intell_Pred) is a well-grounded choice for predicting intelligence-related candidate proteins in most organisms across biological kingdoms. In particular, we assessed seismonastic movements and associate learning in plants and evaluated the proteins involved using Intell_Pred. Proteins related to seismonastic movement and associate learning showed high percentages of similarities with intelligence-related proteins. Our findings lead us to believe that Intell_Pred can help identify the intelligence-related proteins and their classes using a given protein/transcript sequence.

## Introduction

Efforts at defining intelligence have long been attempted, however, there are still challenges in specifying a precise definition. To date, many definitions have been proposed for intelligence which involve a confusing mix of concepts [1]. Intelligence may be defined in terms of speed of learning, the sum of one’s knowledge, and the ability to communicate through language. Thinking and abstract reasoning appear important for intelligence, and one may claim that creative problem solving is at the root of intelligence [2]. However, according to another characterization, learning and memory capacity define intelligence. Accordingly, learning and memory are considered critical components of intelligence [3]. Memory marks a certain experienced event to enable the organism to react faster and more appropriately in similar cases. Learning is tightly associated with memory since it is defined as the capability for more adaptive reactions [4]. Memory and learning are the result of the modification of behavioral patterns and make it possible for an organism to sense, monitor, evaluate and make decisions. Therefore, detailed insight into memory and learning will be useful for studying the various aspects of intelligence.

Proteins are the major constituents of a cell and play vital roles in different parts of biological processes and behavior. Memory which forms the basis of learning and intelligence is among the biological processes that depend on covalent modifications of preexisting proteins (short-term memory) or require regulated gene transcription and translation (long-term memory) [4–8]. Moreover, epigenetic processes resulting from gene expression and thus protein synthesis have been proposed to play a role in cognitive processes [9]

The number of unannotated proteins in databases like UniProtKB/TrEMBL is large; a number that continues to rise daily. Annotation of such proteins not only requires experimental equipment, It is also quite laborious, expensive and time-consuming. Furthermore, in order to investigate intelligence from a holistic perspective, evaluation of a couple of proteins in different organisms cannot deliver the goods. Computational and high-throughput *in silico* approaches can prove helpful when it comes to studying proteins involved in different biological processes such as memory and learning, critical traits of intelligence [10].

Several homologous detecting-based tools, such as BLAST and PSI-BLAST, are being customarily employed for predicting the potential function of unannotated proteins [11–14]. But there are difficulties in using these methods in terms of time and memory and also in cases where low similarities between input and target sequences exist [15,16]. Therefore, many efforts have been dedicated to developing efficient computational approaches to address these problems. In this regard, machine-learning (ML) based methods have been successfully developed, as alternative computational approaches for the prediction of the potential function of proteins, using various features of proteins as input [11,17–21]. To the best of our knowledge, there is still no implementation of ML methods for identifying intelligence-related proteins. Therefore, it would be highly interesting to develop an algorithm that can directly discriminate between intelligence-related proteins and other protein classes.

Support-vector-machine (SVM) is a supervised learning model and a powerful algorithm in pattern recognition and data classification [22]. The basic idea of SVM for binary classification is firstly to transform the non-linearly separable training data into a higher dimensional feature space by a nonlinear mapping, which is realized by defining proper kernel function (different kernel functions can be used). Then, it constructs a separating hyper-plane as the decision surface in such a way that the margin of separation between two classes is maximized [22]. SVM is one of the widely used ML-based algorithms, based on supervision learning model, which has been successfully applied in various trajectories, including prediction of fertility-related proteins [21,23], lipid metabolism-related proteins [11], protein-protein interactions [24–26], drug discovery and prediction of potentially druggable proteins [20,27], discriminating toxic peptides from nontoxic peptides and proteins [28,29] and prediction of protein structure [30,31]. SVM is more precise than the other ML methods [32] and having the ability to recognize subtle patterns in a variety of complex datasets [33]. Moreover, high accuracy, as well as power of high dimensional data handling, makes SVM the most promising classifier in different realms of science [11,34,35]. Inspired by the wide application of ML methods, in this study a novel predictor, Intell_Pred, was constructed using SVM for identifying proteins related to intelligence. Hence, the main objective of this study was to develop this software, which can discriminate between intelligence-related proteins (in two classes including memory and learning) and non-related intelligence using various features extracted from the already annotated intelligence-related proteins. Based on a series of recent studies [36–38] and our previous studies [11,17,21] Chou’s 5-step rules [39] were applied to develop the software, which make all the processes more understandable and transparent. For the sake of illustration, we make use of the developed software to identify and evaluate specific intelligence-related proteins in plants. Our methodology may nonetheless be applied more broadly.

## Material and methods

**Figure 1.**
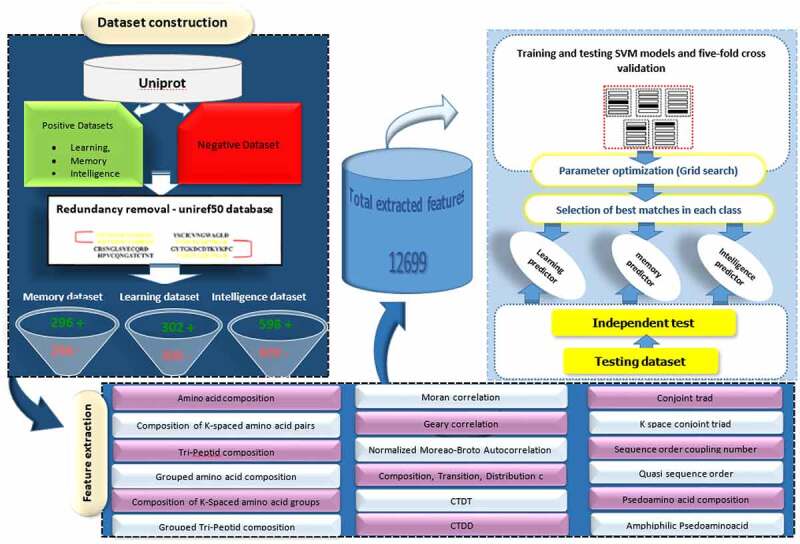
Pipeline of developing SVM-based models for Intell_Pred software.

In order to develop Intell_Pred, Chou’s five-step rules [40] were applied as follows: 1) Creation of an appropriate benchmark dataset for testing and training of the prediction, 2) Usage of an effective formulation for representing the samples that can truly reflect their intrinsic correlation with the target to be predicted, 3) Introduction or development of a powerful algorithm to implement the prediction, 4) Proper interpretation of cross-validation tests to evaluate prediction accuracy; and 5) Establishment of a user-friendly or public available tool for the developed predictor. These steps are presented in Figure 1.

### Benchmark datasets

According to previous studies [17,39,41,42] for developing an efficient statistical predictor, it is of crucial importance to construct a high-quality benchmark dataset containing training and testing datasets. To do so, our previous method was applied to construct the datasets [17,21]. Briefly, through searching the reviewed proteins in UniProtKB database with gene ontology terms “learning”, “memory” and “learning or memory”, protein sequences were collected and were considered as positive datasets related to learning, memory and intelligent classes, respectively. To remove the homologous sequences with sequence identity above 50% and avoid overestimation of the prediction accuracy of the developed models, Uniref50 database was used. Then, only protein sequences with the length <6000 or >60 amino acids were kept. Finally, protein sequences containing non-canonical amino acids such as B, X, and Z were removed. By doing so, a total of 598, 302, 296, proteins were obtained for “intelligence”, “learning” and “memory” classes, respectively.

To create the negative datasets, the UniProtKB database was depleted by a comprehensive search of all keywords implying intelligence functionality (including “not learning”, “not behaviour”, “not memory”, “not habituation” and “not cognition” as well as “not signaling”) and the same filtering steps that were used for positive datasets. In the next step, all domains of the proteins in both datasets were extracted through Pfam database and were compared. Proteins in the negative dataset were removed if they had a common domain with the proteins in positive datasets. Reliability of this method has been reported in previous studies [17,21] and ensures that a negative dataset is constructed by non-intelligence-related proteins exclusively. Eventually, a number of 338,139 non-intelligence-related protein sequences were obtained as negative dataset.

Models trained on very skewing datasets (unbalanced datasets) lead to the production of biased results toward the class with larger number of sequences (negative datasets) [43]. In our case, the positive and negative datasets were extremely unbalanced and the negative dataset was substantially greater than that of positive ones. For this reason, and with an eye to minimizing the bias caused by the skewed datasets and handle the class unbalance problem of the positive and negative datasets, a random sampling solution approach was applied. To this end, we created a balanced benchmark dataset by selecting the protein samples randomly from the negative dataset at the same size of the positive datasets (without replacement). On the other hand, by this method, we cannot cover all of the diversity in the negative dataset. In other words, a random sample of the negative dataset may not be enough to assess the generalized predictive ability of the trained model. To overcome the issue several different negative samples were combined with a positive dataset. Previous studies confirmed the reliability of this approach to consider all the diversity in the negative dataset [17,21]. To do this, five samples were randomly obtained from the negative dataset (at the same number of the positive dataset and without replacement). In the next step, the positive dataset was combined with each of the negative samples, separately. In result, five benchmark datasets were created with the same positive proteins and different negative protein sequences. This approach led to the construction of 15 datasets related to intelligence, memory and learning classes, each with five datasets. More details about this approach can be found in [17,21]. Finally, 20% of each dataset was randomly selected as an independent dataset, while the other 80% was used as the training dataset for training SVM models. For readers’ convenience, protein accession numbers of these datasets are provided in Supplementary File S1.

### Extraction of protein features

Protein feature extraction is a crucial step of concept learning process in which using mathematical model amino acid sequences are represented into a fixed feature vector. Therefore, in addition to a reliable and stringent benchmark dataset, the perfect formulation of the protein sequences is necessary for the development of a useful statistical predictor. This step enables us to find informative physicochemical features from the primary protein sequences, taking into account both the protein constituents and amino acids relative positions, which can be effectively used in the classification step. In general, protein features can be divided into two categories including structure-based features and sequence-based features. Given the difficulty of solving an experimental protein structure, there are significantly more protein sequences in Uniprot than known structures in the Protein Data Bank (PDB) [44]. Therefore, sequence-based features are more suitable for analyzing the proteins without known structures [45]. In this context, pseudo amino acid composition (PseAAC or Chou’s PseAAC) has been proposed as an effective approach for representing and distinguishing protein sequences of different functional profiles [11,17,21,41,46,47]. In this line, according to the concept of general PseAAC, a python programmed tool, iLearn [48], has been developed to calculate these structural and physicochemical features of proteins and peptides. Here, this software was applied to convert each protein sequence into a vector of 12,669 dimensions. These features were belonged to 15 different features descriptors (Table 1). The efficiency of these features for developing the high-quality ML predictors is demonstrated in our previous studies [11,17,21]. Furthermore, inspired by the successes of applying these features to develop the tools for predicting the protein sequences, they have been wildly used in most of the computational proteomics studies [49–53] and many fields of genome analysis [54]. Detailed description of the protein features is provided in Supplementary File S2.

**Table 1.** Summary of 12,669 extracted protein features that were used in SVM-based model development. More details are provided in Supplementary File S2.

	Feature name	Complete feature name	Number of features	Reference
1	AAC	Amino Acid Composition	20	[62]
2	CKSAAP	Composition of k-spaced Amino Acid Pairs	2400	[63]
3	TPC	Tri-Peptide Composition	8000	[62]
4	GAAC	Grouped Amino Acid Composition	5	[64]
5	CKSAAGP	Composition of k-spaced Amino Acid Group Pairs	150	[65]
6	GTPC	Grouped Tri-Peptide Composition	125	[65]
7	Moran	Moran correlation	240	[66–68]
8	Geary	Geary correlation	240	[68]
9	NMBroto	Normalized Moreau-Broto Autocorrelation	240	[67]
10	CTD	Composition /transition/Distribution	39	[69–72]
11	CTDC	CTDT	39	[65]
12	CTDD	CTDD	195	[65]
13	CTriad	Conjoint Triad	343	[73]
14	KSCTriad	k spaced conjoint Triad	343	[65]
15	SOCNumber	Sequence order coupling Number	60	[65]
16	QSOrder	Quasi sequence order	100	[65]
17	PAAC	Pseudo Amino Acid Composition	50	[74,75]
18	APAAC	Amphiphilic Psedudo Amino Acid Composition	80	[75,76]

### Support vector machine (SVM)

As the third step of the Chou’s five-step rules, an algorithm is required to operate the training and predicting. Being intensively and successfully used in many different areas of bioinformatics [55] and computational biology [11,17,21,56–59], SVM is a supervised learning model and a powerful algorithm in pattern recognition and data classification [22]. The basic idea of SVM for binary classification is firstly to transform the non-linearly separable training data into a higher dimensional feature space by a nonlinear mapping, which is realized by defining proper kernel function (different kernel functions can be used). Then, it constructs a separating hyper-plane as the decision surface in such a way that the margin of separation between two classes is maximized. The more detailed information on SVM has been elaborated in [22]. Moreover, for a brief formulation of SVM and how it works, see the [60,61].

In the present study, the positive and negative training datasets were inputted into LIBSVM package (version 3.22) for building a predictive model. The predictive efficiency of SVM models mainly depends on a proper setting of kernel function that maps the input vectors to a high-dimensional feature space [77]. Here, the often-used radial basis function (RBF) was applied as kernel function to obtain the best classification hyper-plane, owing to its wide and successful applications in most of the exiting studies related to ML methods [11,21,78–80]. Furthermore, the two important uncertain parameters in RBF kernel, the regularization parameter C and the kernel width parameter γ, were optimized via an unbiased parameter selection using the grid search approach. This method can guarantee the unbiased results by selecting SVM parameters in a cross-validating manner (five-fold cross validation) [21]. Here to efficiently develop an SVM model to not only able to predict whether a protein is intelligence-related but also able to predict its class (memory or learning), a two-layer classification framework was constructed. First, intelligence dataset (including memory and learning datasets) was used to train SVM model for the first layer. The trained model in the first layer generally is served to predict a query protein sequence as intelligence or non-intelligence related proteins. In the second layer, the SVM models were trained with memory and learning training datasets, separately as binary predictor. A protein is applied as input to the second layer, if it be recognized as intelligence-related protein in the previous layer. The second layer determines the potential of that protein to be related to memory or learning classes. The final decision of a query sequence which is intelligence-related and determination of its class was made according to the probability obtained by SVM. The software considers a probability score >0.5 to designate putative intelligence-related proteins and their classes (memory or learning).

### Model performance evaluation

We need to answer two questions for measuring the prediction quality of the developed models. The first one is: What indices should be used to measure the model’s quality? And the other one is: What test method should be used to score them? The questions are answered as follows, respectively.

The performance of the developed models evaluated by five indices, including accuracy (ability of the predictor to differentiate intelligence from non-intelligence), sensitivity (ability to correctly identify intelligence) and specificity (ability to correctly identify non-intelligence) and Matthew’s correlation coefficient (MCC, a correlation coefficient between the observed and predicted binary classifications). Since the conventional equations of these measures are not quite intuitive, the proposed formulae by Chen et al [81] (based on general formulations of these metrics) are given below:

(1.1) $$Sn=1-\frac{N_{-}^{+}}{N^{+}}\;\;\;\;\;\;\;\;\;\;\;0\;\leq Sn\leq \;1$$

(1.2) $$Sp=1-\frac{N_{+}^{-}}{N^{-}}\;\;\;\;\;\;\;\;\;\;\;0\;\leq Sp\leq \;1$$

(1.3) $$Acc=1-\frac{N_{-}^{+}+N_{+}^{-}}{N^{+}+\;N^{-}}\;\;\;\;\;\;\;\;\;\;\;\;0\;\leq Acc\leq \;1\;$$

(1.4) $$MCC=\;\frac{1-\;\left(\frac{N_{-}^{+}}{N^{+}}+\frac{N_{+}^{-}}{N^{-}}\;\right)}{\sqrt{\left(1+\;\frac{N_{+}^{-}+N_{-}^{+}}{N^{+}}\;\right)\left(1+\;\frac{N_{-}^{+}+N_{+}^{-}}{N^{-}}\right)}}\;\;\;\;\;\;\;-1\;\leq MCC\leq \;1$$

In the above formulas, $N^{+}$ and $N^{-}$ denote the total number of the positive and negative protein sequences investigated, whereas $N_{-}^{+}$ and $N_{+}^{-}$ is the number of the intelligence/non-intelligence proteins incorrectly predicted to be non-intelligence /intelligence, respectively.

To examine the ability of the predictive model in identifying intelligence protein sequences, five-fold cross-validation was carried out (as test method) on training datasets. To do so, the training datasets were divided into five equally sized subsets. In the next step, each of the subsets was singled out one by one and tested by the SVM trained with the remaining four subsets. These processes were repeated five times since each subset was used as testing set once. Finally, the five validation results were combined to generate a single accuracy. In addition, independent datasets (or testing datasets, which is definitely blind to the training dataset) were applied to allay the concerns of over-fitting the performance of the constructed model to the training dataset.

### Software development

To enhance the practical usability and publicly available software for practical applications, a two-layer classifier software called Intell_Pred (intelligence prediction) has been provided freely at https://github.com/mrb20045/Intell_Pred. The software allows the users to predict intelligence-related proteins along with their potential classes (memory or learning). The software can be used by a wide variety of researchers with limited knowledge of the SVM computing environment, which needs simply upload sequence(s) in FASTA format for prediction. A key point of the Intell_Pred is accepting mRNA transcripts as input, in addition to amino acids sequences. Intell_Pred benefits from TransDecoder tool (version 3.0.1, http://transdecoder.sourceforge.net) to identify candidate open reading frames (ORFs) within mRNA transcripts. Then, the predicted amino acid sequences by TransDecoder, automatically input to the SVM models for predicting their potential as intelligence-related proteins as well as their classes. It is merit to note that the software only needs a FASTA file (DNA/protein) as input and performs all the processes automatically. Finally, it will return the name of the inputted sequence(s), predicted results along with probability scores (0≤ score ≥1) for every sequence.

## Results and Discussions

With the explosive growth of biological sequences in the post-genomic era, a growing demand has raised for efficient methods to annotate these sequences. Previous studies declared that forming memory and learning relies on cross talk between genetic and epigenetic variations [82]. Learning involves a series of molecular changes including regulation of gene transcription. The basis of the long-term memory form, when a number of genetic modifications are maintained in post-mitotic cells throughout life [83]. Considering proteins as molecular fossils and the final products of gene regulation [15], in-detail study of proteins involved in intelligent behaviors can light the way to discover the mechanism of forming memory and learning and finally to investigate intelligence in individuals. In this study, for the first time, an SVM-based two-layer classifier, Intell_Pred, designed for predicting the potential of unannotated proteins, which most likely to be suspected in intelligence.

### Model development

Here, the benchmark datasets were used with 50% identity based on the previous studies, which noted that datasets with higher identity or high homologous sequences increase overestimation risk of prediction [21]. All accession numbers of the used proteins are represented in Supplementary File S1 (a positive dataset combined with five different negative datasets in each class). To maximize the estimated overall accuracy by SVM, penalty parameter C and γ (width or number of attributes) of RBF were adjusted and computed by grid search strategy based on five-fold cross validation test. As a result, the penalty parameter C was set at 10 for all datasets and the width γ ranged from 0.001 to 0.003 for different datasets. The four metrics achieved by the proposed classifier via the five-fold cross-validation and independent datasets testing along with optimized γ values for each benchmark datasets are given in Tables 2–4.

**Table 2.** Accuracy metrics of train and test datasets for intelligence model. The optimum γ parameter value of kernel function of SVM was chosen using a grid-search technique based on five-fold cross-validation.

	Dataset 1		Dataset 2		Dataset 3		Dataset 4		Dataset 5		Average	
γ	0.001		0.003		0.001		0.003		0.001		-	
	Train	Test	Train	Test	Train	Test	Train	Test	Train	test	Train	Test
Accuracy (%)	89.58	88.75	88.59	87.55	89.75	87.6	88.99	87.97	88.57	86.72	89.09	87.71
Sensitivity (%)	88.06	87.80	87.20	84.73	89.50	87.10	87.72	87.10	87.38	88.70	87.97	87.08
Specificity (%)	91.43	89.74	90.25	90.91	90.22	88.14	90.45	88.89	89.92	84.92	90.45	88.52
MCC (%)	79.33	77.52	77.30	75.35	79.60	75.21	78.06	75.95	77.23	73.54	78.3	75.51

**Table 3.** Accuracy metrics of train and test datasets for memory model. The optimum γ parameter value of kernel function of SVM was chosen using a grid-search technique based on five-fold cross-validation.

	Dataset 1		Dataset 2		Dataset 3		Dataset 4		Dataset 5		Average	
γ	0.001		0.003		0.001		0.003		0.001		-	
	Train	Test	Train	Test	Train	Test	Train	Test	Train	test	Train	Test
Accuracy (%)	86.77	88.24	87.15	91.6	87.92	92.44	85.27	88.14	88.60	93.39	87.14	90.76
Sensitivity (%)	86.10	88.33	85.70	93.10	86.32	94.74	83.43	86.89	87.60	93.55	85.83	91.32
Specificity (%)	88.52	88.14	89.38	90.16	89.85	90.32	88.51	89.47	89.94	93.22	89.24	90.26
MCC (%)	74.08	76.46	74.69	83.24	75.87	84.98	71.25	76.31	77.33	86.76	74.64	81.55

**Table 4.** Accuracy metrics of train and test datasets for learning model. The optimum γ parameter value of kernel function of SVM was chosen using a grid-search technique based on five-fold cross-validation.

	Dataset 1		Dataset 2		Dataset 3		Dataset 4		Dataset 5		Average	
γ	0.003		0.003		0.003		0.003		0.003		-	
	Train	Test	Train	Test	Train	Test	Train	Test	Train	test	Train	Test
Accuracy (%)	85.30	88.43	86.76	87.6	86.96	88.33	84.27	89.17	87.78	87.5	86.21	88.2
Sensitivity (%)	83.15	86.15	84.19	83.82	84.28	84.85	81.09	85.07	85.74	85.71	83.69	85.12
Specificity (%)	88.63	91.07	90.05	92.45	90.67	92.59	89.37	94.34	90.36	89.47	89.81	91.98
MCC (%)	71.19	77.01	73.89	75.69	74.43	77.05	69.50	78.87	75.83	75.09	72.96	76.74

SVM model achieved an average prediction accuracy of 89.09% at the first layer, which determines the protein can be related to intelligence or not (intelligence as general class) (Table 2). The model maintained its predictive properties (with accuracy of 87.71%), when independent datasets were applied, which shows that Intell_Pred is highly promising. The same situation was observed for the models in the second layer (learning and memory models), which determine the potential of the passed protein from the first layer to be related to memory or learning classes (Tables 3 and 4). From these results, it can be found that sensitivity, specificity and accuracy of different datasets are very close, meaning that there was no bias in classification in all models. In other words, there is an equal chance of identifying the intelligence and non-intelligence-related proteins, correctly. To further emphasize the effectiveness of the proposed models, roughly 20% of each dataset was kept in each class for independent evaluation of the final models (testing datasets). Similar results were obtained based on the independent blind test, as the positive datasets could be discriminated from negative datasets with acceptable accuracy metrics in all classes. This suggests a promising capability and robustness of the proposed method for identifying the intelligence-related proteins and their classes (Tables 2–4).

### Evaluation of Intell_Pred with new positive and negative proteins

To the best of our knowledge, Intell_Pred is the first predictor ever developed for intelligence and non-intelligence proteins classification. Hence, there is not any possibility to compare its performance with its counterparts for exactly the same purpose. But our method achieved a high accuracy in comparison with those reported in most of the previous studies [11,21,49,51,78–81] in other areas. However, to further challenge the proposed method for predicting intelligence-related proteins, new negative and positive datasets were constructed and evaluated using Intell_Pred. To this end, 10 negative datasets (each dataset contains 1000 protein sequences) were constructed through selecting randomly from negative datasets, as none of these proteins were appeared during training or testing the SVM models. For new positive dataset, UniProtKB database was searched (since after Intell_Pred had been developed) and new annotated proteins as intelligence-related ones were found through comparing with our primary positive dataset. In total, 85 and 84 new proteins were found, which were belonged to memory and learning classes, respectively. Results of evaluation of the new negative and positive datasets using Intell_Pred are displayed in Tables 5 and 6, respectively. Also, complete results of evaluating the new negative and positive proteins using Intell_Pred are provided in Supplementary Files S3 and S4, respectively.

**Table 5.** Summary results of evaluation of the 10,000 new negative protein sequences by Intell_Pred.

	Score* >0.5		Score >0.75	Score >0.90		
	Number	Percent	Number	Percent	Number	Percent
Intelligence	1058	10.58	612	6.12	347	3.47
Learning	844	8.44	587	5.87	308	3.08
Memory	815	8.15	484	4.84	219	2.19

* Score indicates the estimated score for a protein to be intelligence-related in the first layer and its score in the second layer to be involved in learning or memory. For example, out of 10,000 negative proteins, only 1058 proteins were detected to be intelligence-related proteins. Higher scores are more reliable.

**Table 6.** Summary results of evaluation of the new annotated protein sequences related to intelligence in Uniprot database by Intell_Pred (169 protein sequences were evaluated including 85 and 84 proteins belong to memory and learning classes, respectively) .

	Score* >0.5		Score >0.75		Score >0.90	
	Number	Percent	Number	Percent	Number	Percent
Intelligence	163	96.45	159	94.08	137	81.07
Learning	81**	96.42	77	91.66	71	84.52
Memory	81 ***	95,329	79	92.94	69	81.17

* Score indicates the estimated score for a protein to be intelligence-related in the first layer and its score in the second layer to be involved in learning or memory. For example, out of 169 evaluated proteins, 163 proteins were detected to be intelligence-related proteins. Higher scores are more reliable. ** Out of 84 proteins that were belonged to learning class. *** Out of 85 proteins that were belonged to memory class.

Interesting results were obtained after evaluation of the new negative and positive protein sequences. Out of 10,000 negative protein sequences, only 1,058 (10.58%) sequences were predicted as intelligence related. But, if score >0.90 considered, only 347 (3.47%) sequences were predicted as intelligence related (Table 5). Out of 169 positive protein sequences 163 (96.45%) sequences were predicted as intelligence related. Of these, 137 (81.07%) sequences were predicted as intelligence related when score >0.90 was considered (Table 6). These findings indicated that larger scores are more reliable, but it may cause to miss some real intelligence related proteins, if score >0.90 be considered. Hence, score >0.75 can be suggested as a reliable choice to balance the sensitivity and specificity for discriminating intelligence and non-intelligence related sequences.

### Plant intelligence and candidate proteins

In this study, a molecular approach was adopted to figure out to which extent seismonastic responses to mechanical stimulation relate to memory and learning. In particular, proteins of *Arabidopsis thaliana* involved in processes related to seismonastic movement mechanisms e.g., proteins involved in pulvinus activation, turgor pressure and leaf movement; cytochalasin b-related protein, the motor cells-related proteins, charge amplification-related proteins, osmotic pressure-related proteins [84] were extracted from the Uniprot database and evaluated using Intell_Pred. As our results reveal, 84.61, 80.76 and 77.07% of the proteins related to seismonastic movement mechanisms were predicted to be involved in intelligence, and learning and memory, respectively (Table 7, Supplementary File S5).

**Table 7.** Summary results of evaluation of the plant candidate proteins using Intell_Pred.

Intelligent attribute	Related proteins	Number of proteins	Intelligence			Learning			Memory		
			≥0.5*	≥0.75	≥0.9	≥0.5	≥0.75	≥0.9	≥0.5	≥0.75	≥0.9
Associate learning in pea	Phototropins	18	17	16	13	17	15	9	17	12	4
Semimonastic movements	Pulvinus activation, … **	52	44	33	21	42	35	20	38	25	10

*Predicted scores. ** Turgor pressure, Leaf movement, Cytochalasin b-related protein, Motor cells related proteins, Charge amplification related protein, Osmotic pressure.

In like vein, proteins related to phototropins were extracted from the Uniprot database, as it was reported that associate learning of the sort that pea plants appear to exhibit [although see 85, 86, and 87, for exchanges on contradictory evidence for the existence of associative learning in plants] [85–88] is related to phototropin function [89]. In total, 18 proteins were found and were evaluated using Intell_Pred. Interestingly, 17 proteins (94.44%) were predicted as intelligence-related proteins, as 16 proteins showed score >0.75. Most of these proteins had score >0.75 in the memory class too (Table 7). The results indicated that tropistic responses triggered by an air flow mediated by the function of phototropin, as observed in Gagliano et al.’s pea plant learning experiments, is an intelligent behavior (72). The results also indicate that intelligence can be the result of memory and learning (4). Complete results of evaluating of these proteins using Intell_Pred are provided in Supplementary Files S5.

Our findings are consistent with the working hypothesis that plants are able to learn and develop memory. Learning, memory and intelligent behavior may not require brain cephalization or otherwise neural network of the sort found in animals. The way forward to sift through the complexity of plant intelligence studies is the integration of the information from several areas within the plant sciences, including molecular and cellular studies of signal transduction, plant physiology, ecology, bioinformatics and molecular biology [90].

## Conclusion

In this study a two-layer SVM-based classifier (Intell_Pred), developed to identify intelligence-related proteins and their classes (memory or learning), that uses complete structural feature of proteins. Different accuracy metrics demonstrated that Intell_Pred can effectively distinguish between intelligence-related and non-intelligence related proteins. Considering that proteins, as molecular fossils, are the final products of gene regulation, identification of the specific intelligence-related proteins may provide a clue for paving the way to unraveling the plant sort of intelligence. In this way, studying in detail the proteins involved in intelligent behavior can ease the discovery of the mechanism that underlie the formation of memory and learning.

From a computational point of view, it is noteworthy that although the prediction of intelligence-related proteins achieves acceptable results, there is room for improvement, as the number of protein sequences for intelligence and their classes is effectively growing on a daily basis. We envisage that Intell_Pred will be a powerful tool for detecting novel intelligence-related proteins in the future.

## Supplementary Material

Supplemental Material
