# Supplementary material for: Identification of intelligence-related proteins through a robust two-layer predictor
Source: Commun Integr Biol. 2022 Nov 15;15(1):253–64. doi: 10.1080/19420889.2022.2143101 (PMC9673931; doi:10.1080/19420889.2022.2143101)
Supplement: Supplemental Material [file KCIB_A_2143101_SM5825.zip › supplement/Supplementary File S2.docx]

**Supplementary File S2. Description of applied structural and physicochemical protein features.**

**Amino Acid Composition (AAC)**

The Amino Acid Composition (AAC) encoding (Bhasin and Raghava, 2004) calculates the frequency of each amino acid type in a protein or peptide sequence. The frequencies of all 20 natural amino acids (i.e. “ACDEFGHIKLMNPQRSTVWY”) can be calculated as:


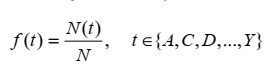


where N(t) is the number of amino acid type t, while N is the length of a protein or peptide sequence.

**Composition of k-spaced Amino Acid Pairs (CKSAAP)**

The CKSAAP feature encoding calculates the frequency of amino acid pairs separated by any k residues (k = 0, 1, 2, … , 5. The default maximum value of k is 5) (Chen, et al., 2009; Chen, et al., 2007a; Chen, et al., 2007b; Chen, et al., 2008). Taking k = 0 as an example, there are 400 0-spaced residue pairs (i.e., AA, AC, AD,…, YY.). Then, a feature vector can be defined as:


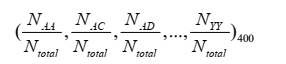


The value of each descriptor denotes the composition of the corresponding residue pair in the protein or peptide sequence. For instance, if the residue pair AA appears m times in the protein, the composition of the residue pair AA is equal to m divided by the total number of 0-spaced residue pairs (Ntotal) in the protein. For k = 0, 1, 2, 3, 4 and 5, the value of Ntotal is P – 1, P – 2, P – 3, P – 4, P – 5 and P – 6 for a protein of length P, respectively. An illustrated example of this encoding scheme (k=0) is provided in the following Figure S1.


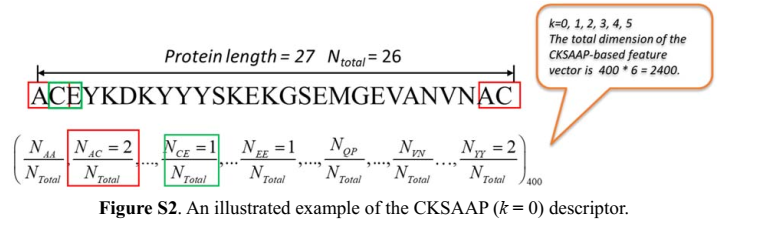


**Figure S1.** An illustrated example of the CKSAAP (k=0) descriptor.

**Tri-Peptide Composition (TPC)**

The Tripeptide Composition (TPC) (Bhasin and Raghava, 2004) gives 8000 descriptors, defined as:
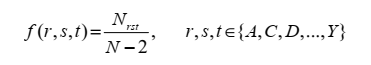


where Nrst is the number of tripeptides represented by amino acid types r, s and t.

**Grouped Amino Acid Composition (GAAC)**

In the GAAC encoding, the 20 amino acid types are further categorized into five classes according to their physicochemical properties, e.g. hydrophobicity, charge and molecular size (Lee, et al., 2011b). The five classes include the aliphatic group (g1: GAVLMI), aromatic group (g2: FYW), positive charge group (g3: KRH), negative charged group (g4: DE) and uncharged group (g5: STCPNQ). GAAC descriptor is the frequency of each amino acid group, which is defined as:


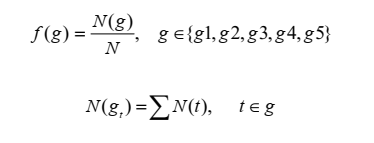


where N(g) is the number of amino acids in group g, N(t) is the number of amino acid type t, and N is the length of the protein/peptide sequence.

**Composition of k-Spaced Amino Acid Group Pairs (CKSAAGP)**

The Composition of k-Spaced Amino Acid Group Pairs (CKSAAGP) is a variation of the CKSAAP descriptor, which is our own proposal. It calculates the frequency of amino acid group pairs separated by any k residues (the default maximum value of k is set as 5). Taking k = 0 as an example, there are 25 0-spaced group pairs (i.e., g1g1, g1g2, g1g3, … g5g5). Thus, a feature vector of CKSAAGP can be defined as:


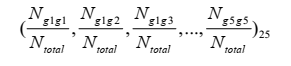


The value of each descriptor denotes the composition of the corresponding residue group pair in a protein or peptide sequence. For instance, if the residue group pair g1g1 appears m times in the protein, the composition of the residue pair g1g1 is equal to m divided by the total number of 0- spaced residue pairs (Ntotal) in the protein. For k = 0, 1, 2, 3, 4 and 5, the values of Ntotal are P – 1, P – 2, P – 3, P – 4, P – 5 and P – 6 respectively, for a protein of length P.

**Grouped Tri-Peptide Composition (GTPC)**

The Grouped Tri-Peptide Composition encoding is also a variation of TPC descriptor, which generates 125 descriptors, defined as:


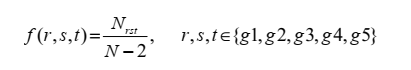


where Nrst is the number of tripeptides represented by amino acid type groups r, s and t. N is the length of a protein or peptide sequence.

**Moran correlation (Moran)**

The autocorrelation descriptors are defined based on the distribution of amino acid properties along the sequence (Feng and Zhang, 2000; Horne, 1988; Sokal and Thomson, 2006). The amino acid properties used here are different types of amino acids index, which is retrieved from the AAindex Database (Kawashima, et al., 2008) available at http://www.genome.jp/dbget/aaindex.html/. The eight indices ‘CIDH920105', 'BHAR880101', 'CHAM820101', 'CHAM820102', 'CHOC760101', 'BIGC670101', 'CHAM810101', 'DAYM780201' are used (Xiao, et al., 2015). An illustrated example of this amino acid physicochemical properties from the AAIndex database is provided in Figure S2.


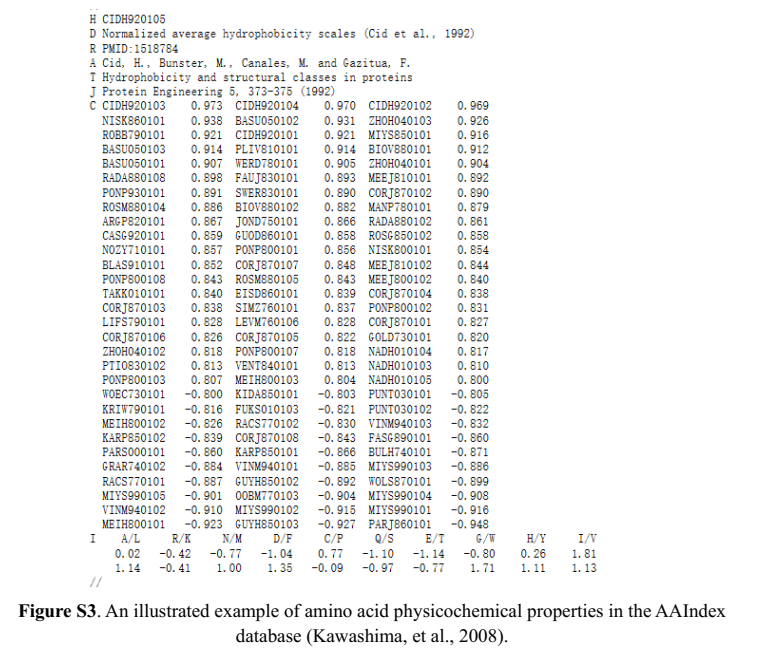


**Figure S2.** An illustrated example of amino acid physicochemical properties in the AA Index.

All the amino acid indices are centralized and standardized prior to the calculation:


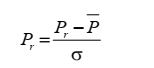


Where $\bar{P}$ is the average of the properties of the 20 amino acids and σ is the standard deviation of the properties of the 20 amino acids. $\bar{P}$ and σ can calculated as follow:


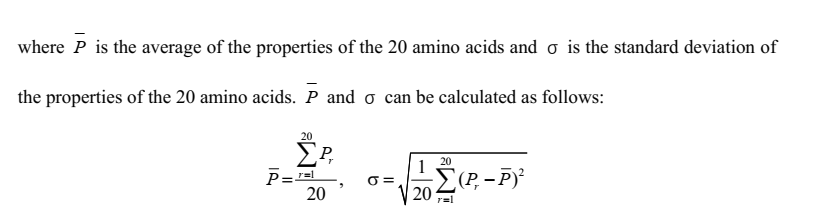


The Moran autocorrelation descriptors (Feng and Zhang, 2000; Lin and Pan, 2001) can thus be defined as:


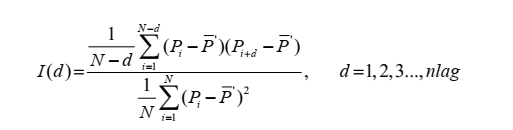


where d is the lag of the autocorrelation, nlag is the maximum value of the lag (default value: 30), Pi and Pi+d are the properties of the amino acids at positions i and i + d, respectively. ܲ′ is the average of the considered property P over the entire sequence of length N and is calculated as:


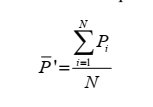


**Geary correlation (Geary)**

The Geary autocorrelation descriptors (Sokal and Thomson, 2006) for a protein or peptide sequence are defined as:


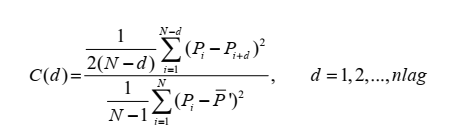


where d, P, Pi and Pi+d, nlag have the same definitions as described above.

**Normalized Moreau-Broto Autocorrelation (NMBroto)**

The Moreau-Broto autocorrelation descriptors (Horne, 1988) are defined as follows:


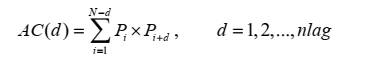


The normalized Moreau-Broto autocorrelation descriptors are thus defined as:


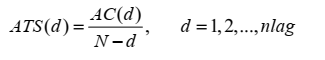


**Composition/Transition/Distribution (CTD)**

The Composition, Transition and Distribution (CTD) features represent the amino acid distribution patterns of a specific structural or physicochemical property in a protein or peptide sequence (Cai, et al., 2003; Cai, et al., 2004; Dubchak, et al., 1995; Dubchak, et al., 1999; Han, et al., 2004). 13 types of physicochemical properties have been previously used for computing these features. These include hydrophobicity, normalized Van der Waals Volume, polarity, polarizability, charge, secondary structures and solvent accessibility. These descriptors are calculated according to the following procedures: (i) The sequence of amino acids is transformed into a sequence of certain structural or physicochemical properties of residues; (ii) Twenty amino acids are divided into three groups for each of the seven different physicochemical attributes based on the main clusters of the amino acid indices of Tomii and Kanehisa (Tomii and Kanehisa, 1996). The groups of amino acids are listed in the below Table.


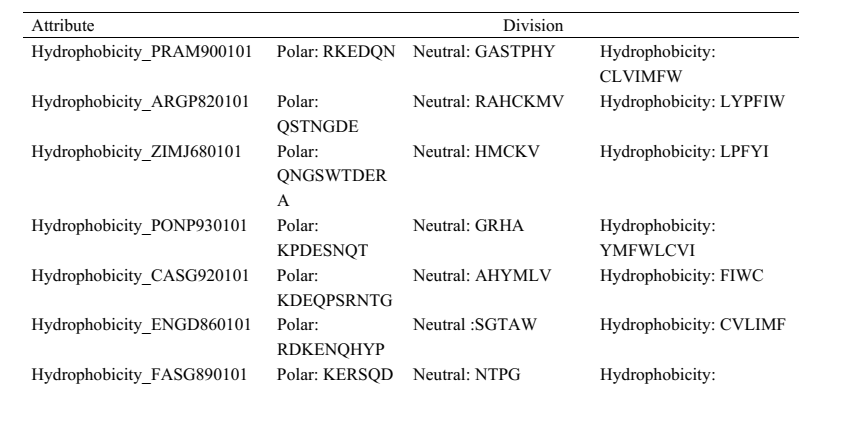

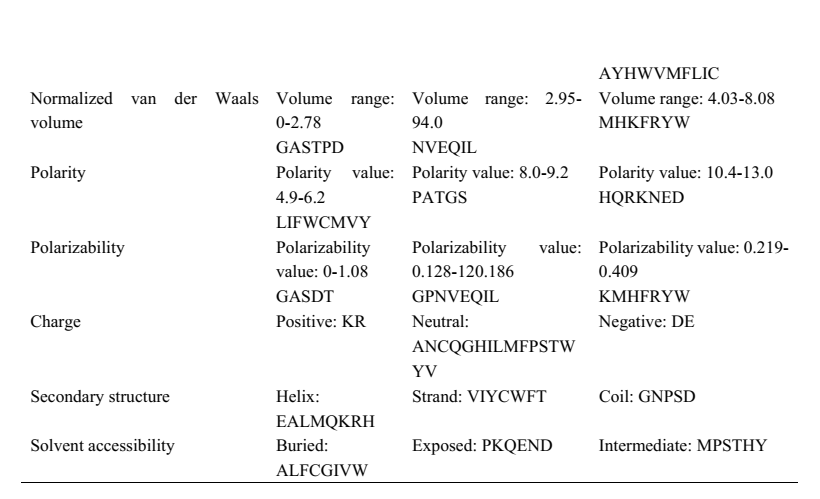


**CTDT**

The Transition descriptor T also consists of three values (Dubchak, et al., 1995; Dubchak, et al., 1999): A transition from the polar group to the neutral group is the percentage frequency with which a polar residue is followed by a neutral residue or a neutral residue by a polar residue. Transitions between the neutral group and the hydrophobic group and those between the hydrophobic group and the polar group are defined in a similar way. The transition descriptor can then be calculated as:


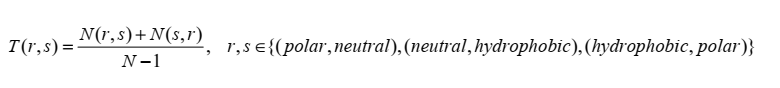
where N(r,s) and N(s,r) are the numbers of dipeptides encoded as “rs” and “sr” respectively in the sequence, while N is the length of the sequence. An illustrated example of this encoding scheme is provided in the following Figure S3.


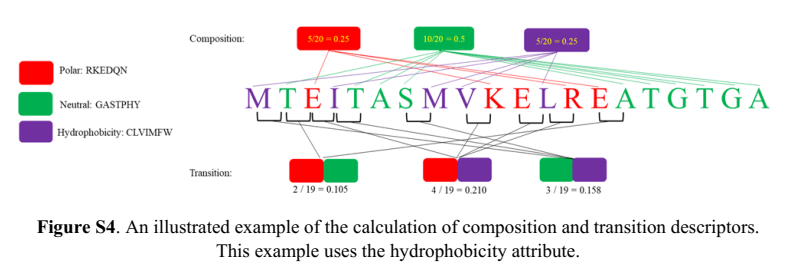


**Figure S3**. An illustrated example of the calculation of composition and transition descriptors. This example uses the hydrophobicity attribute.

**CTDD**

The Distribution descriptor consists of five values for each of the three groups (polar, neutral and hydrophobic) (Dubchak, et al., 1995; Dubchak, et al., 1999), namely the corresponding fraction of the entire sequence, where the first residue of a given group is located, and where 25, 50, 75 and 100% of occurrences are contained. For example, we start with the first residue up to and including the residue that marks 25/50/75/100% of occurrences for residues of any given group and then we simply divide the position of this residue by the length of the entire sequence.

**Conjoint Triad (CTriad)**

The Conjoint Triad descriptor (CTriad) considers the properties of one amino acid and its vicinal amino acids by regarding any three continuous amino acids as a single unit (Shen, et al., 2007). First, the protein sequence is represented by a binary space (V, F), where V denotes the vector space of the sequence features, and each feature (Vi) represents a sort of triad type; F is the number vector corresponding to V, where fi, the value of the i-th dimension of F, is the number of type Vi appearing in the protein sequence. For the amino acids that have been catalogued into seven classes, the size of V should be equal to 7 ⅹ 7ⅹ 7=343. Accordingly, i = 1, 2, 3, …, 343. An illustrated example of this encoding scheme is provided in the following Figure S4. In principle, the longer a protein sequence, the higher the probability to have larger values of fi, confounding the comparison of proteins with different lengths. Thus, we define a new parameter, di, by normalizing fi with the following equation:


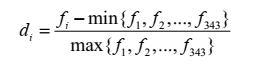


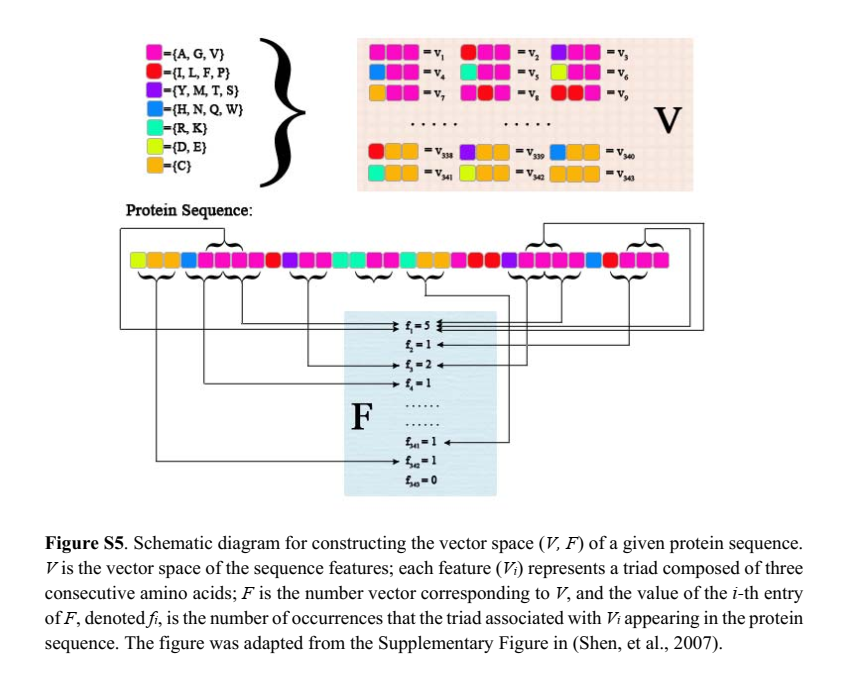
 **Figure S4.** Schematic diagram for constructing the vector space (V, F) of a given protein sequence. V is the vector space of the sequence features; each feature (Vi) represents a triad composed of three consecutive amino acids; F is the number vector corresponding to V, and the value of the i-th entry of F, denoted fi, is the number of occurrences that the triad associated with Vi appearing in the protein sequence. The figure was adapted from the Supplementary Figure in (Shen, et al., 2007).

**k-Spaced Conjoint Triad (KSCTriad)**

The k-Spaced Conjoint Triad (KSCTriad) descriptor is based on the Conjoint CTriad descriptor, which not only calculates the numbers of three continuous amino acid units, but also considers the continuous amino acid units that are separated by any k residues (The default maximum value of k is set to 5). For example, AxRxT is a 1-spaced triad. Thus, the dimensionality of the KSCTriad encoded feature vector is 343 (k+1). An illustrated example of this encoding scheme is provided in the following Figure S5.


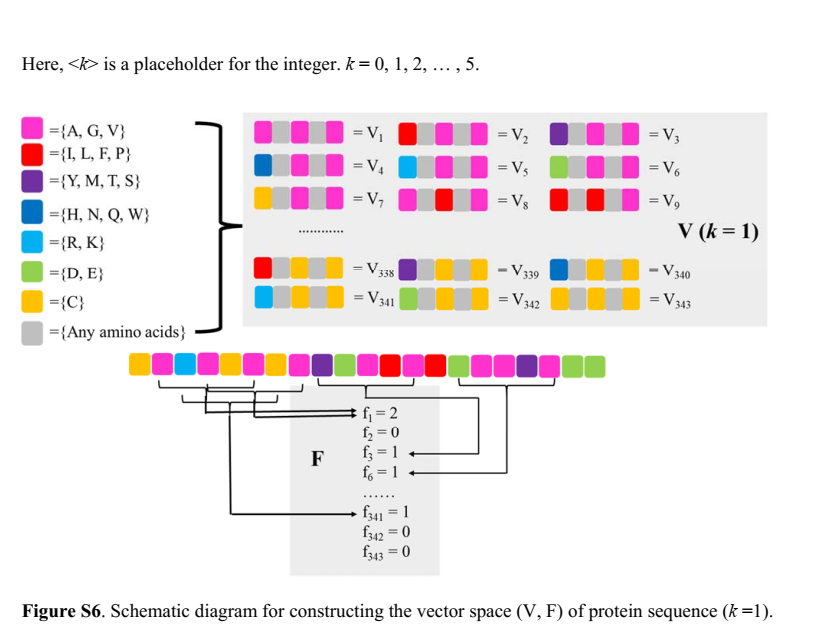


**Figure S5.** Schematic diagram for constructing the vector space (V, F) of protein sequence (k=1).

Sequence-Order-Coupling Number (SOCNumber). The d-th rank sequence-order-coupling number is defined as:


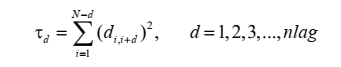


where di,i+d is the entry in a given distance matrix describing a distance between the two amino acids at position i and i + d, nlag denotes the maximum value of the lag (default value: 30) and N is the length of a protein or peptide sequence. As distance matrix both the Schneider-Wrede physicochemical distance matrix (Schneider and Wrede, 1994) used by Kuo-Chen Chou, and the chemical distance matrix by Grantham (Grantham, 1974) are used. Accordingly, the descriptor dimension will be nlagⅹ 2. The quasi-sequence-order descriptors described next also utilizes the two matrices. An illustrated example of this encoding scheme is provided in the following Figure S6.


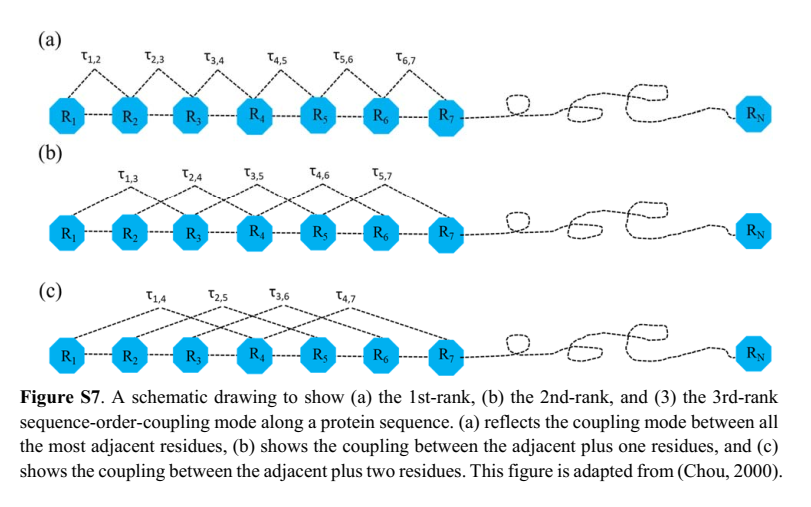


**Figure S6.** A schematic drawing to show (a) the 1st-rank, (b) the 2nd-rank, and (3) the 3rd-rank sequence-order-coupling mode along a protein sequence. (a) reflects the coupling mode between all the most adjacent residues, (b) shows the coupling between the adjacent plus one residue, and (c) shows the coupling between the adjacent plus two residues. This figure is adapted from (Chou, 2000).

**Quasi-sequence-order (QSOrder)**

For each amino acid type, a quasi-sequence-order descriptor can be defined as:


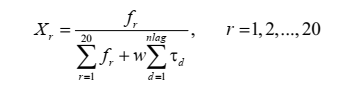


where fr is the normalized occurrence of amino acid type r and w is a weighting factor (w = 0.1), nlag and ߬ௗ have the same definitions as described above. These are the first 20 quasi-sequence order descriptors. The other 30 quasi-sequence-order descriptors are defined as:


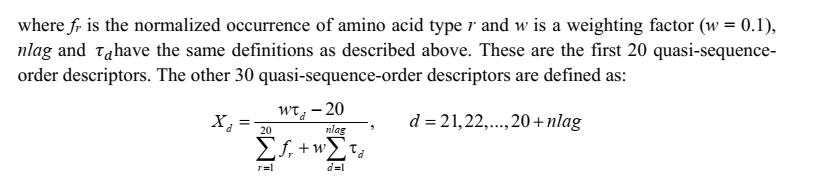


**Pseudo-Amino Acid Composition (PAAC)**

This group of descriptors has been proposed in (Chou, 2001; Chou, 2005). Let
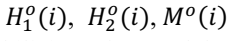
 for i = 1, 2, 3, … 20 be the original hydrophobicity values, the original hydrophilicity values and the original side chain masses of the 20 natural amino acids, respectively. They are converted to the following quantities by a standard conversion:


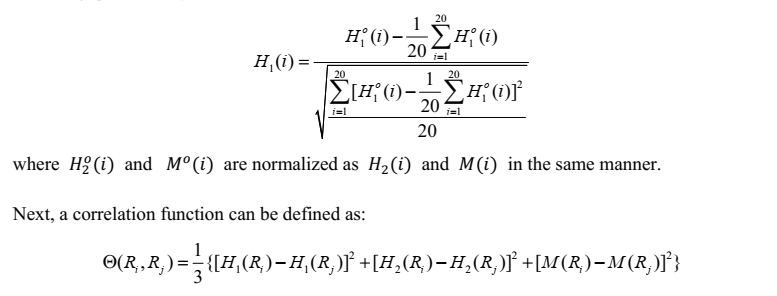


Where $H_{2}^{0}(i)$ and $M^{0}(i)$ are normalized as $H_{2}(i)$ and M(i) in the same manner.

Next, a correlation function can be defined as:


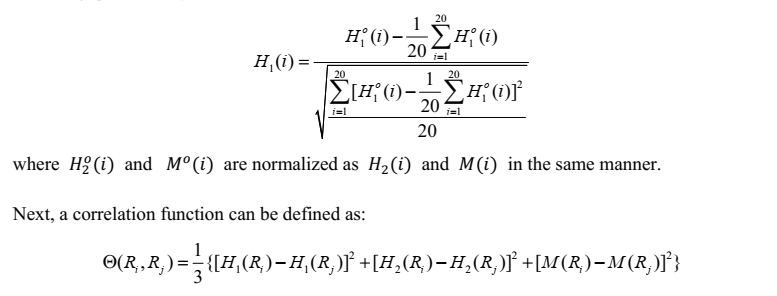


This correlation function is actually an averaged value for the three amino acid properties: hydrophobicity value, hydrophilicity value and side chain mass. Therefore, we can extend this definition of correlation function for one amino acid property or for a set of n amino acid properties. For one amino acid property, the correlation can be defined as:


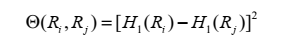


where H(Ri) is the amino acid property of amino acid Ri after standardization. An illustrated example of the correlation function is provided in the following Figure S7.


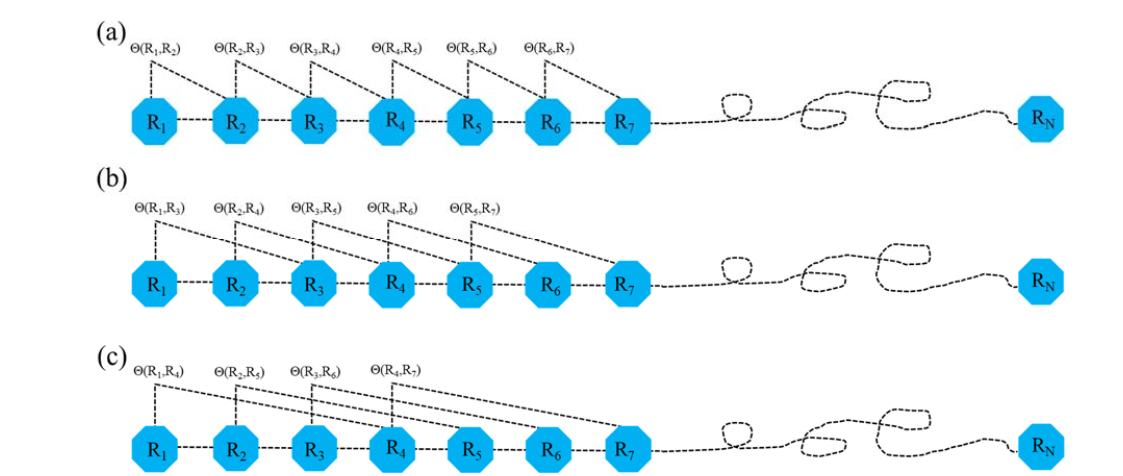


Figure S7. A schematic drawing to show (a) the first-tier, (b) the second-tier, and (3) the third-tier
sequence order correlation mode along a protein sequence. (a) Reflects the coupling mode between
all the most adjacent residues, (b) shows the coupling between the adjacent plus one residues, and
(c) shows the coupling between the adjacent plus two residues. This figure is adapted from (Chou,
2001) for illustration purposes.

For a set of *n* amino acid properties, it can be defined as:


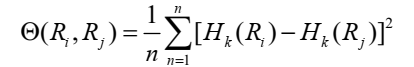


Where *Hk(Ri)* is the *k*-th property in the amino acid property set for amino acid *Ri*.
A set of descriptors called sequence order-correlated factors are defined as:


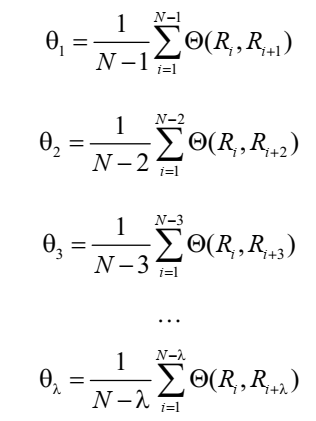


Where λ (λ < *N*) is an integer parameter to be chosen. Let *fi* be the normalized occurrence
frequency of amino acid *i* in the protein sequence. Then, a set of 20 + λ descriptors called the
pseudo-amino acid composition for a protein sequence can be defines as:


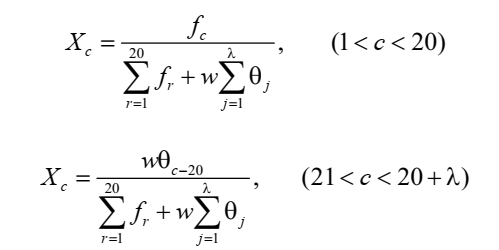


Where *w* is the weighting factor for the sequence-order effect and is set to *w* = 0.05 in *iFeature* as
suggested by Chou *et al*. (Chou, 2001).

**Amphiphilic Pseudo-Amino Acid Composition (APAAC)**

Amphiphilic Pseudo-Amino Acid Composition (APAAC) was proposed in (Chou, 2001; Chou, 2005). The definition of this set of features is similar to the PAAC descriptors. Using H1(i) and H2(j) as previously defined, the hydrophobicity and hydrophilicity correlation functions are defined as:


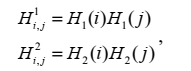


respectively. An illustrated example of the correlation functions is provided in the following Figure S8. Thus, sequence order factors can be defined as:


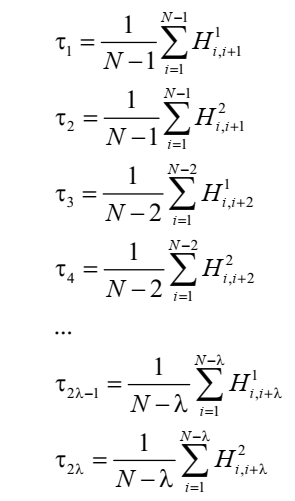


Then, a set of descriptors, called Amphiphilic Pseudo-Amino Acid Composition (APAAC), is
defined as:


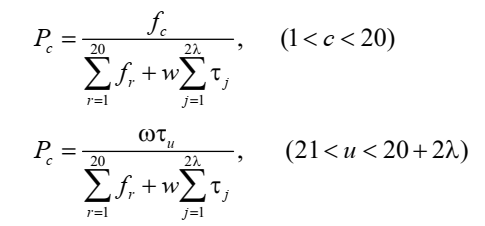


Where *w* is the weighting factor. In *iFeature* this factor is set to *w* = 0.5 as described in Chou’s work (Chou, 2001).


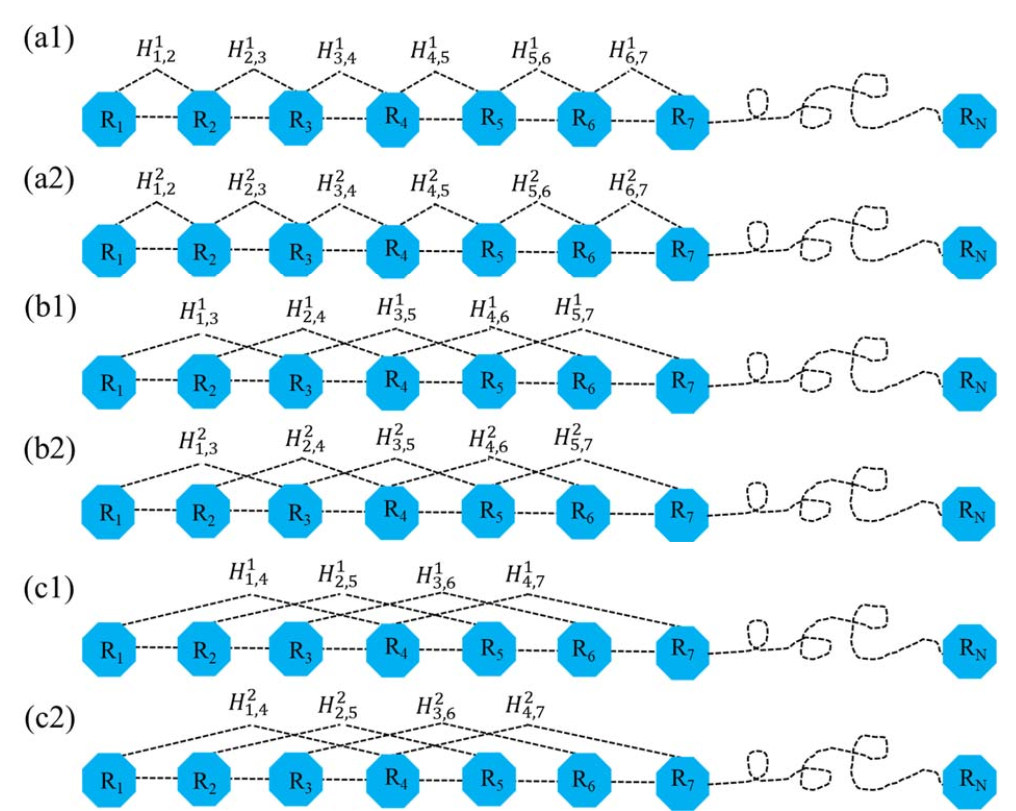


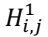
**Figure S8**. A schematic diagram to show (a1/a2) the first-rank, (b1/b2) the second-rank and (c1/c2)
the third-rank sequence-order-coupling mode along a protein sequence through a
hydrophobicity/hydrophilicity correlation function, where and
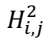
 are given by the
aforementioned equation. Panels (a1/a2) reflects the coupling mode between the most adjacent
residues, panels (b1/b2) show the coupling between the adjacent plus one residue, and panels
(c1/c2) shows the coupling between the adjacent plus two residues. This figure is adapted from
(Chou, 2005) for illustration purposes.

1. Chen, Z.; Zhao, P.; Li, F.; Leier, A.; Marquez-Lago, T. T.; Wang, Y.; Webb, G. I.; Smith, A. I.; Daly, R. J.; Chou, K.-C., iFeature: a python package and web server for features extraction and selection from protein and peptide sequences. *Bioinformatics* **2018,** 34, (14), 2499-2502.
