# Supplementary material for: Identification of intelligence-related proteins through a robust two-layer predictor
Source: Commun Integr Biol. 2022 Nov 15;15(1):253–64. doi: 10.1080/19420889.2022.2143101 (PMC9673931; doi:10.1080/19420889.2022.2143101)
Supplement: Supplemental Material [file KCIB_A_2143101_SM5825.zip › supplement/Supplementary legends.docx]

**Supplementary Files**

**Supplementary File S1:** Accession number list of the positive and negative protein sequences that were used for developing Intell_Pred

**Supplementary File S2:** Description of applied structural and physicochemical protein features.

**Supplementary File S3:** Detailed results of evaluation of the 10000 new negative protein sequences by Intell_Pred.

**Supplementary File S4:** Detailed results evaluation of the new annotated protein sequences related to intelligence in Uniprot database by Intell_Pred (169 protein sequences were evaluated including 85 and 84 proteins belong to memory and learning classes, respectively).

**Supplementary File S5:** Detailed results evaluation of the plant candidate proteins using Intell_Pred, the 70 protein sequences were evaluated including 18 and 52 proteins belong to associate learning in pea and semimonastic movements, respectively).
